# Cell-in-cell phenomena across the tree of life

**DOI:** 10.1038/s41598-024-57528-7

**Published:** 2024-03-29

**Authors:** Stefania E. Kapsetaki, Luis H. Cisneros, Carlo C. Maley

**Affiliations:** 1 Arizona Cancer Evolution Center, Arizona State University, Tempe, AZ USA; 2 Biodesign Center for Biocomputing, Security and Society, Arizona State University, Tempe, AZ USA; 3 Department of Biology, School of Arts and Sciences, Tufts University, Medford, MA USA; 4 School of Life Sciences, Arizona State University, Tempe, AZ USA; 5 Biodesign Center for Mechanisms of Evolution, Arizona State University, Tempe, AZ USA; 6 Center for Evolution and Medicine, Arizona State University, Tempe, AZ USA

**Keywords:** Social evolution, Cancer, Cell biology, Oncology

## Abstract

Cells in obligately multicellular organisms by definition have aligned fitness interests, minimum conflict, and cannot reproduce independently. However, some cells eat other cells within the same body, sometimes called cell cannibalism. Such cell-in-cell events have not been thoroughly discussed in the framework of major transitions to multicellularity. We performed a systematic screening of 508 articles, from which we chose 115 relevant articles in a search for cell-in-cell events across the tree of life, the age of cell-in-cell-related genes, and whether cell-in-cell events are associated with normal multicellular development or cancer. Cell-in-cell events are found across the tree of life, from some unicellular to many multicellular organisms, including non-neoplastic and neoplastic tissue. Additionally, out of the 38 cell-in-cell-related genes found in the literature, 14 genes were over 2.2 billion years old, i.e., older than the common ancestor of some facultatively multicellular taxa. All of this suggests that cell-in-cell events may have originated before the origins of obligate multicellularity. Thus, our results show that cell-in-cell events exist in obligate multicellular organisms, but are not a defining feature of them. The idea of eradicating cell-in-cell events from obligate multicellular organisms as a way of treating cancer, without considering that cell-in-cell events are also part of normal development, should be abandoned.

## Introduction

Evolutionary transitions in individuality, by definition, refer to a collective alteration after which the units within the “higher-level” individual have aligned fitness interests, minimal conflict amongst themselves, and cannot reproduce independently anymore. Despite the constraints imposed by this definition, there are eusocial species and obligate multicellular organisms with: (1) chimerism, where genetically distinct units of the “higher-level” individual have unaligned fitness interests (unless both genetically distinct units pass to the next generation)^1^; and (2) genetically related units of the group eating each other, and thus displaying significant relational conflict. In the cellular context relevant to multicellularity, this second type of phenomenon is generally called “cell-in-cell events”.

There are many categorizations of cell-in-cell phenomena mostly based on functional traits^2^ (Supplementary Table 2; also see glossary in Fais and Overholtzer^3^). Cell-in-cell phenomena have been previously classified based on whether the internalized cell was dead (phagocytosis), or alive (pathogenic phagocytosis, entosis, emperipolesis, etc.)^2,4,5^. Cell-in-cell phenomena have also been defined based on the type of interacting cells: identical (entosis, homogenous cannibalism) or different (emperipolesis, heterogenous cannibalism)^6–8^, and in the case of tumors, tumor cell cannibalism^9,10^. Cell-in-cell phenomena have also been classified according to their initial internalization stage: resembling endocytosis (cannibalism, phagoptosis, and enclysis) or invasion of the ‘prey’ cell into the host (entosis and emperipolesis)^11^. Another classification depends on whether the whole (e.g., phagocytosis) or part(s) (e.g., in trogocytosis, nibbling, cannibalism, and partial phagocytosis) of the prey cell are internalized^12,13^. Other times, cell-in-cell phenomena have been classified entirely by the outcome of the cell-in-cell event: death of the host and/or death of the internalized cell, division of the internalized cell, or exit of the internalized cell from the host cell^14–16^.

There are several open questions in the field of cell-in-cell phenomena. Why are there “cell-in-cell” phenomena? Conspecific cell-in-cell phenomena can be explained by several fitness benefits to the host cell, prey cell, and potential intracellular viruses (Supplementary Table 4A). Where across the tree of life do we see cell-in-cell phenomena? Are they associated with a ‘selfish’ phenomenon like cancer? Were cell-in-cell phenomena required for, or just incidental to, the transition to obligate multicellularity?

In this review, we focus on the role of cell-in-cell phenomena in the context of evolutionary transitions in multicellularity^17,18^. Specifically, we include only phenomena of cells internalizing whole cells and not just parts of other cells. We first identify cell-in-cell phenomena across the tree of life and propose a classification based on social evolution. Second, we summarize whether conspecific cell-in-cell phenomena are associated with cancer according to the literature. Third, we examine the association and origins of cell-in-cell phenomena in relation to the evolution of multicellularity, by: (i) categorizing cell-in-cell phenomena according to the combination of their degree of ‘selfishness’ and their degree of multicellularity (starting from unicellularity, facultative multicellularity, to obligate multicellularity); and (ii) phylogenetically dating the origin of cell-in-cell-related genes.

## Results

**Figure 1:**
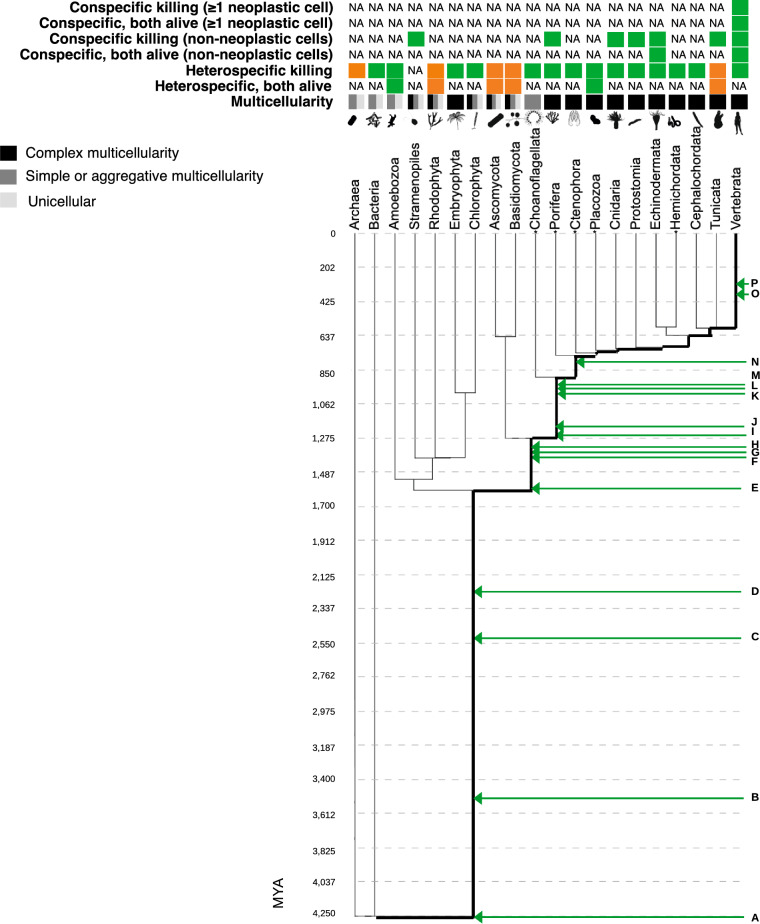
Phylogenetic tree of multicellularity and cell-in-cell phenomena. Adjusted using multicellularity data from^19–21^. We have highlighted in bold the line of how we found the position of each human gene in time from Vertebrates down to the closest common ancestor of all taxa shown in this phylogenetic tree. Made with NCBI Common Tree^22^, iTOL^23^ (version 6.7.4), and PhyloPic (phylopic.org) (version 2.0). Branch lengths show the approximate ages (MYA) of each branch from TimeTree. (**A**) AlyA plays a role in lysozyme activity; FspA is a possible receptor or regulator in the folate-sensing pathway. (**B**) SLC11A1 is involved in killing bacteria inside the cell. (**C**) FAT1 mediates cell–cell adhesion and can bind to β-catenin. (**D**) ACTB is involved in cell–cell adhesion; CDC42 downregulation controls phagocytosis and entosis; PRKAA1, PRKAB1,2, and PRKAG1,2,3 are involved in AMPK which is required for entosis; RAB7A is a phagocytic marker; LYST is involved in lysosome maturation. (**E**) TM9SF4 is associated with tumour cell cannibalism; CYBB is involved in killing bacteria inside the cell. (**F**) KRAS is an entotic marker. (**G**) CD163 is a macrophage marker involved in cell-in-cell phenomena; MSR1 is a macrophage marker. (**H**) CTSG regulates pathogen killing in neutrophils. (**I**) RHOA is involved in entosis and phagocytosis; DIAPH1 is involved in cell-tension pathways during entosis. (**J**) WASF1 is involved in phagosome maturation. (**K**) MYH1,2 and CTNNB1 are involved in cell–cell adhesion during entosis; ADGRE1 is a phagocytic marker; EZR is involved in cell cannibalism and heterotypic cell-in-cell phenomena; CTNNA2 is required for entosis; TP53: enhances engulfment processes; CD68 is a phagocytic and cannibalistic marker; LAMP1 is a phagolysosomic marker; CD36 mediates phagocytosis. (**L**) CDH1,2,3 are cell-in-cell and entotic markers. (**M**) SELL is involved in heterotypic cell-in-cell phenomena. (**N**) LYZ is a cannibalistic marker. (**O**) CD2 is involved in heterotypic cell-in-cell phenomena. (**P**) ICAM1 is involved in heterotypic cell-in-cell phenomena. Examples of conspecific killing (≥ 1 neoplastic cell) include entosis, cannibalism, and the host cell not always being an immune cell. Examples of conspecific cell-in-cell phenomena, where both cells remain alive (≥ 1 neoplastic cell) include entosis, emperipolesis, cannibalism, with the host cell not always being an immune cell. Examples of conspecific killing between non-neoplastic cells include phagocytosis, entosis, cannibalism, with the host cell not always being an immune cell. Examples of conspecific cell-in-cell phenomena between non-neoplastic cells, where both cells remain alive, include emperipolesis, with the host cell not always being an immune cell. Phagocytosis, heterotrophy, and bacterivory are examples of heterospecific killing. Phagocytosis and endosymbiosis are examples of heterospecific cell-in cell phenomena, where both cells remain alive. A green box indicates that the specific cell-in-cell category type has been found in that taxon, whereas an orange box indicates no heterospecific or conspecific cell-in-cell phenomena, i.e., researchers searched for heterospecific or conspecific cell-in-cell phenomena in these taxa but did not find such phenomena. NA shows that cell-in-cell phenomena have not been searched for in these taxa by researchers. Detailed information about the genes can be found in Supplementary Table 3. *No cancer-like phenomena reported.

Cell-in-cell phenomena have been found in 16 taxonomic groups across the tree of life. Cell-in-cell phenomena across the seven different phyla examined in our study can be separated into six different categories from the perspective of social evolution (Fig. 1; Supplementary Table 2):

1. Heterospecific killing between non-neoplastic cells where at least one of the resulting cells dies, is the most common phenomenon out of the six categories of cell-in-cell phenomena appearing in all seven examined unicellular, facultative or obligately multicellular phyla (Fig. 1; Supplementary Table 2).
2. Conspecific killing between non-neoplastic cells where at least one of the resulting cells dies is the second most common phenomenon appearing in three out of the seven examined unicellular, facultative or obligately multicellular phyla (Fig. 1; Supplementary Table 2).
3. Heterospecific cell-in-cell phenomena between non-neoplastic cells where both of the cells remain alive have been found in three out of the seven examined unicellular or facultatively multicellular taxa (Fig. 1; Supplementary Table 2).
4. Conspecific cell-in-cell phenomena between non-neoplastic cells where both of the cells remain alive have been found in one out of the seven examined obligately multicellular phyla (echinoderms) (Fig. 1; Supplementary Table 2).
5. Conspecific killing where at least one cell is neoplastic and at least one of the resulting cells dies appears only in one obligately multicellular taxon (vertebrates) (Fig. 1; Supplementary Table 2).
6. Conspecific cell-in-cell phenomena where at least one cell is neoplastic and both of the cells remain alive appears only in one obligately multicellular taxon (vertebrates) (Fig. 1; Supplementary Table 2).

In the domains of archaea and bacteria, only heterospecific killing between non-neoplastic cells has been found in bacteria where at least one of the resulting cells dies. Across the four examined divisions of Ascomycota, Basidiomycota, Chlorophyta, and Rhodophyta, only the Chlorophyta have a form of cell-in-cell event which is heterospecific killing between non-neoplastic cells where at least one of the resulting cells dies. Across the two examined clades of stramenopiles and embryophyta, the former have conspecific killing between non-neoplastic cells where at least one of the resulting cells dies, whereas the latter have heterospecific killing between non-neoplastic cells where at least one of the resulting cells dies. Across the three examined subphyla of tunicates, cephalochordata, and vertebrates, the tunicates have conspecific killing between non-neoplastic cells where at least one of the resulting cells dies, the cephalochordata have heterospecific killing between non-neoplastic cells where at least one of the resulting cells dies, and all cell-in-cell categories exist in vertebrates except for heterospecific cell-in-cell events between non-neoplastic cells where both of the resulting cells remain alive (Fig. 1; Supplementary Table 2).

In many taxa out of the 20 (Fig. 1), many of the above phenomena have not been found or have not been searched for (Supplementary Table 2A). Within vertebrates, cell-in-cell phenomena have been described in eight species (Supplementary Table 2B).

Conspecific cell-in-cell phenomena also occur between non-neoplastic cells and appear in multicellular organisms that have no known cancer or cancer-like development. According to the literature (Supplementary Table 2), there are several examples of cell-in-cell phenomena between non-neoplastic cells. Also, porifera display conspecific cell-in-cell phenomena but have no known cancer-like growth (Supplementary Table 2). An important caveat here is that the fact that no cancer-like phenomena have been reported in these taxa does not mean that they do not get cancer. They may not have been adequately studied yet to know if they can get cancer^19^.

We were able to identify 38 cell-in-cell-related genes, at least 14 of which likely evolved before the origins of obligate multicellularity (Fig. 1). 14 cell-in-cell-related genes originated over 2.2 billion years ago: AlyA, FspA, SLC11A1, FAT1, LYST, ACTB, CDC42, PRKAA1, PRKAB1,2, PRKAG1,2,3, and RAB7A (Fig. 1; A–D). They are related to the AMPK pathway, folate-sensing pathway, cell–cell adhesion, entosis, phagocytosis, intracellular bacterial killing, cell cannibalism, lysozyme activity, and lysosomal maturation (Supplementary Table 3). Specifically, FspA, which is involved in the folate-sensing pathway, is necessary for the sensing of the bacteria *Klebsiella* by *Dictyostelium,* prior to *Dictyostelium* killing the bacteria via phagocytosis^24^. 24 cell-in-cell-related human genes originated after the common ancestor of amoeba and humans. These genes (Fig. 1; E–P) are related to cell-in-cell phenomena such as cell–cell adhesion, entosis, macrophage function, phagocytosis, killing pathogens, phagosome maturation, and cell cannibalism. TM9SF4 is a gene related to tumor cell cannibalism. KRAS is a gene related to entosis. CD163, MSR1, and CTSG are genes related to killing pathogens. DIAPH1 is a gene related to entosis, and RHOA is a gene related to entosis and phagocytosis. Homologs of MYH1,2 and CTNNB1 are involved in cell adhesion during entosis, CDH1,2,3 are involved in entosis, WASF1, ADGRE1, and LAMP1 are related to phagocytosis, EZR is related to cell cannibalism and heterotypic cell-in-cell phenomena, CTNNA2 is required for entosis, TP53 is required for cell engulfment, CD36 is involved in phagocytosis, CD68 is involved in phagocytosis and cell cannibalism, and SELL is related to heterotypic cell-in-cell phenomena. LYZ is a gene related to cell cannibalism. Two cell-in-cell-related human genes, CD2 and ICAM1, originated in the taxon of vertebrates around 313–400 million years ago (Fig. 1; O–P). These genes are involved in entosis, heterotypic cell-in-cell phenomena, and phagosome maturation. No cell-in-cell phenomena have been reported in archaea, red algae, ascomycota, and basidiomycota, even though homologs of 14 cell-in-cell-related genes could be present in the red algae (Fig. 1; A–D) and homologs of 20 cell-in-cell-related genes could be present in the ascomycota and basidiomycota (Fig. 1; A–H). By separating these genes according to their date of origin and the associated cell-in-cell phenomenon (Table 1), we find that not all of these cell-in-cell phenomena (phagocytosis, entosis, cell cannibalism, intracellular bacterial killing) share the exact same genetic architecture at the functional level.

**Table 1:** Cell-in-cell phenomena do not all share the exact same genetic architecture at the functional level.

Cell-in-cell phenomenon	Genes older than 2.2 BYA (14 genes)	Genes younger than 2.2 BYA (24 genes)
Phagocytosis	CDC42, RAB7A	CD163, MSR1, CTSG, RHOA, WASF1, ADGRE1, CD68, LAMP1, CD36
Cannibalism		TM9SF4, EZR, CD68, LYZ
Entosis	CDC42, PRKAA1, PRKAB1,2, PRKAG1,2,3	KRAS, RHOA, DIAPH1, MYH1,2, CTNNB1, CTNNA2, CDH1,2,3
Intracellular bacterial killing	SLC11A1	CYBB, CTSG
Not only specific to the above phenomena (they have a more general cell-in-cell function)	AlyA, FspA, FAT1, ACTB, LYST	CD163, EZR, TP53, CDH1,2,3, SELL, CD2, ICAM1

There are certainly other genes involved in these phenomena. Here we only classify those genes identified in the literature on those phenomena. More details about the genes’ cell-in-cell related function in Supplementary Table 3. BYA: billion years ago.

## Discussion

This study led to four main findings. First, cell-in-cell phenomena are present in at least 16 different taxonomic groups on the tree of life. These cell-in-cell phenomena can be classified into six useful categories from the perspective of social evolution that depend on the degree of relatedness between host and prey (conspecifics or heterospecifics), the outcome of the event (whether both or only one cell remains alive after the cell-in-cell phenomenon), and the type of cells (neoplastic or non-neoplastic cells). Second, conspecific cell-in-cell phenomena occur in neoplastic cells as well as non-neoplastic cells. Third, we did not find any significant association between cell-in-cell phenomena and the levels of multicellularity. Fourth, 38 cell-in-cell-related genes exist across the tree of life, and some of those genes predate the origins of obligate multicellularity.

### Cannibal neoplastic and non-neoplastic cells

Cell-in-cell phenomena happen between neoplastic and non-neoplastic cells. Depending on the details of the cell interaction, the outcome of cannibalism can be either the growth or shrinkage of the neoplasm. Thus, cannibalism cannot be considered a characteristic of only uncontrollably dividing ‘selfish’ neoplastic cells.

Due to their relatively higher nutritional demands, neoplastic cells are often cannibals^25^. Cell-in-cell phenomena can lead to aneuploidy and/or tetraploidy, and more aggressive and invasive tumors^26,27^. Therefore, it is no surprise that cannibalism is more often found in malignant than benign biopsies and urine cytology samples^28,29^, in metastatic tumors than in primary melanoma^30^, in aggressive than in non-aggressive giant cell granuloma^31^, in grade 3 rather than in grade 1 urothelial carcinoma^32,33^, in higher rather than in lower grade breast tumors^34^, and in higher rather than in lower grade superficial papillary cancer^35^. Still, finding cell-in-cell phenomena together with malignancy does not necessarily mean that cell-in-cell phenomena induce malignancy, neither it is sufficient nor necessary for malignancy. In fact, cell-in-cell phenomena may be a mechanism to suppress malignancy. For example, entosis was shown to suppress tumor growth in breast cancer cell lines^36^, and breast cancer cells that have cannibalized mesenchymal stem/stromal cells were shown to enter a dormant state^37^.

Cell-in-cell phenomena occur outside of the context of cancer. *Bdellovibrio* bacteria were observed to enter and kill other Gram-negative bacteria^38^ without any evidence of preference for killing ‘cheating’ bacteria. Poriferan cells perform conspecific cell-in-cell phenomena without any evidence of cancer-like growth (Fig. 1). Additionally, cell-in-cell phenomena are characteristic of non-cancer cell types during development. Sperm and egg can fuse, forming a zygote. Around 5–6 days after the first cell division of a human embryo, some of the mother’s (uterine epithelial) cells enter the fetus (trophectoderm) cells^39^. We can assume that this process reduces the chances of fetal cells being rejected by the mother. In *Caenorhabditis elegans*, endodermal cells eat parts of primordial germ cells^40^. In *Xenopus laevis*, endoderm cells can engulf their own as well as their neighbor’s membrane^41,42^. In *Drosophila*, mice^43^, zebrafish^44,45^, and humans, later in development, multinucleated cells can form when myoblast cells fuse with myoblast cells^46^, osteoclast cells fuse with other osteoclast cells, and hepatocyte cell fuse with other hepatocyte cells^47^. Phagocytosis is also a common mechanism for the immune system to discard pathogenic foreign cells, as well as cells from the same organism that have been programmed for disposal (Supplementary Table 2).

### Origins of cell-in-cell-related genes

We searched for associations between 38 cell-in-cell-related genes and the origins of obligate multicellularity, as well as correlations between the levels of ‘selfishness’ of cell-in-cell phenomena and the level of multicellularity, but did not find any significant associations. This shows that, mechanistically, cell-in-cell phenomena likely predate the evolution of obligate multicellularity. A gene worth mentioning reminds us of Charles Darwin’s saying about how development can teach us about evolution: the transmembrane 9 superfamily protein member 4 gene TM9SF4^48^ in the obligately multicellular humans encodes an ion channel, possibly regulating the pH of intracellular vesicles of malignant cells^49^. Since the origin of this gene, ~ 1.5 billion years ago, another gene phg1A, a homolog of TM9SF4, has been found in facultatively multicellular extant amoeba *Dictyostelium discoideum* playing a role in phagocytosis^9,49,50^. Thus, TM9SF4 and its homolog have a history in cell-in-cell phenomena before and after the evolution of obligate multicellularity.

Cell-in-cell genes might play a role in the connections between carnivory, placental invasiveness, and cancer mortality risk. In univariate and multivariate model comparisons, Dujon et al.^51^ found that species with a diet of mostly vertebrates and with an endotheliochorial (where some of the mother’s uterine epithelial cells have entered trophectoderm cells of the blastocyst via entosis^39^) or haemochorial placental type (the most invasive type of placenta) have a higher cancer mortality risk than species with an epitheliochorial type of placenta (the least invasive type of placenta).

### Limitations

Despite observations in the literature that cell-in-cell phenomena exist in both benign and malignant neoplasms as well as during normal development, cell-in-cell phenomena have not been searched for in many species across the tree of life.

In this article we summarize the cell-in-cell-related functions of genes found in humans, cats, dogs, mice, zebrafish, turtles, *Drosophila*, *Dictyostelium*, and bacteria, as mentioned in the literature (Supplementary Table 3). In the case of cell-in-cell-related genes found in non-human organisms, we also mention the cell-in-cell related function of the human homologue gene (Supplementary Table 3). For all cell-in-cell-related genes found in humans, cats, dogs, mice, zebrafish, turtles, *Drosophila*, *Dictyostelium*, and bacteria, we also mention the age of the human homologue dating back to the last common ancestor between humans and the corresponding current non-human taxon that carries that gene. Importantly, if a gene originated before the origins of a taxon (where we currently observe cell-in-cell-related genes and phenomena), it does not mean that the original gene had a cell-in-cell-related function. The cell-in-cell-related function could have evolved after the origins of the gene on the genome. Therefore, with the date of origin of a cell-in-cell-related gene we do not imply that this was the date of origin of its function, but the date of origin of that gene sequence.

For building Fig. 1, we have used the NCBI Taxonomy^22^ and the TimeTree^52,53^ databases. Neither of these tools, however, have adopted the most updated topology of the eukaryotic tree of life^54–56^. For example, in the NCBI taxonomic tree the Amoebozoa are in a different supergroup^57^, whereas the Amoebozoa are now grouped in the newly defined Amorphea supergroup^54–56^. In order to maintain consistency between tools leading to estimated evolutionary timings from TimeTree, we opted to maintain the NCBI tree as our reference. Our general results are not significantly affected by this choice, but future phylogenetic studies ought to consider adopting an updated eukaryotic tree of life (which likely would be implemented in a new version of the NCBI taxonomy database).

Understanding what drives the forms of cell-in-cell phenomena that suppress tumor growth versus those that enhance tumor growth could help the management of cancer. For example, following a calorie-restricted diet^58^ or using caloric restriction mimetics in clinical trials^59^ may not necessarily be a successful strategy to treat cancer considering that starvation drives cell-in-cell phenomena (Supplementary Table 4), and cell-in-cell phenomena can lead to both tumor suppression and tumor progression^26,36,60^. Therefore, understanding the difference in conditions that determine one outcome versus the other would be of principal importance, and potentially of benefit for treatment based on a precision medicine approach. Interestingly, exosomes can be ingested by cancer cells^61^. The process of internalization of exosomes into cells occurs in a similar way to endocytosis^62^ or phagocytosis^63^. However, serving cannibalistic cancer cells a ‘ticking bomb’ of exosomes full of drugs may not be a successful anticancer strategy either, as these cells can also ‘spit out’^64^ exosomes. Mapping cell-in-cell-related genes onto phylogenies and homology families using appropriate criteria of evolutionary relatedness that take into account cell-in-cell phenomena, disentangling the corresponding genetic networks, ascertaining the role of possibly antagonistic pleiotropic cell-in-cell-related genes, their expression patterns before and after reproductive age, and exploring the micro-/macro-environmental triggers that might change the risk of cell-in-cell events suppressing or promoting cancer, will be major cuts through the ‘Gordian knot’ in understanding the evolutionary history of multicellularity, cancer, and cell-in-cell phenomena across species.

### Open question: connection between microscopic and macroscopic cannibalistic phenomena

There may also be connections between microscopic and macroscopic cannibalistic phenomena. Factors that drive cannibalism in the microscopic world (micrometer scale) may also drive cannibalism in the macroscopic world (centimeter or meter scale). Examples of such analogies can be seen in Supplementary Table 4. Under starvation, both cells and obligate multicellular organisms (e.g., honeybees, mantises, and mice) perform cannibalism. Mammals with large litter sizes also cannibalize their young in periods of food scarcity^65^. Under attack by the enemy (immune system or specific tribe), cancer cells and humans (respectively) cannibalize their enemy. Upon landing in a new environment, entotic uterine cells and cannibalistic beetles are more likely to survive (Supplementary Table 4). However, no one has yet quantitatively estimated all the different factors (natural selection, random genetic drift, mutation, migration) that may drive cannibalistic processes both at the microscopic and macroscopic scales.

## Conclusion

Overall, this study is the first to systematically analyze cell-in-cell phenomena across the tree of life. Here we provide a classification of cell-in-cell phenomena using a social evolution perspective, in the context of evolutionary transitions. This work highlights how cell-in-cell phenomena can be organized by the degree of relatedness, survival or death outcome of the interacting cells, and ‘selfish’ overall/cancerous behavior, in terms of the fitness outcome (survival), of the interacting cells. This study also highlights that cell-in-cell phenomena are not only found among cancer cells but also among benign neoplastic and normal cells. Cell-in-cell phenomena are not a definitive feature of obligate transitions to multicellularity, given that cell-in-cell phenomena have been found in organisms that are facultatively multicellular and have no multicellular ancestry. Thus, the prerequisite for minimal conflict in the definition of major transitions in multicellularity refers to ‘conflict’, as in the presence of cell-in-cell phenomena within a broad range of multicellular organisms, and ‘minimal’, as in not all cells of multicellular organisms eat each other.

## Methods

In order to find records of cell-in-cell phenomena across the tree of life we used the Arizona State University Library One Search tool, which includes the ASU Library catalog and other online search engines, such as Google Scholar, Mendeley, and JSTOR. We searched for articles using the following keywords:

(entos?s OR “homotypic cell cannibalism” OR “cell cannibalism” OR emperitos?s OR enclysis) AND (vertebrat* OR urochord* OR cephalochord* OR echinoderm* OR protostom* OR cnidaria* OR ascomycot* OR basidiomycot* OR amoebozoa* OR embryophyt* OR chlorophyt* OR rhodophyt* OR stramenopila* OR bacter*).

During January 2022 to January 2023, we also searched for articles that mentioned “*phagocytosis*” and cases of cannibalism specifically in the taxa shown in the phylogenetic tree of Aktipis et al^19^. This led to 352 articles. When an article mentioned “*entos?s*”, “*homotypic cell cannibalism*”, “*cell cannibalism*”, “*emperitos?s*”, and referenced another article, we searched for the original publication and included the original publication in Supplementary Table 2 if the content was relevant to our search. This method has been previously used when conducting systematic reviews^66^. Searching back for such citations added 156 articles to our list. For a more objective assessment of the literature, two of the authors read several of the articles independently. L.H.C. assessed 249 articles, reading from the oldest to the most recent, and S.E.K. assessed 360 articles, reading from the most recent to the oldest. Out of these, a total of 101 articles were assessed by both L.H.C. and S.E.K. We removed patents, web resources, books, newsletter articles, government documents, book chapters, newspaper articles, conference proceedings, reviews, and non-English articles, which led to 337 articles for further assessment. We also excluded 222 articles that turned out to be about irrelevant topics, review articles that directed us to more relevant articles (specifically to articles with original data), articles with no information about cell-in-cell phenomena in specific taxa, or with no information regarding the fate of the engulfed or host cell in terms of one or both remaining alive after the cell-in-cell event (Supplementary Table 1).

Our final assessment included 115 articles (Supplementary Table 1; Supplementary Figure). We collected the following information from these 115 articles: (1) whether the cell-in-cell phenomenon was between heterospecifics or conspecifics; (2) whether the host cell engulfed the whole prey cell; (3) whether both cells remained alive after the cell-in-cell event or at least one cell died; (4) whether both cells were non-neoplastic cells or at least one cell was a neoplastic cell; and (5) the specific taxon of the host cell. We included this information in our across-species comparisons (Supplementary Table 2; Fig. 1).

Our third aim was to examine associations between cell-on-cell phenomena and the evolution of multicellularity across 20 taxa based on previous scales of multicellularity^19,20,67^. We performed the following ordinal categorical scaling: we categorized taxa in Supplementary Table 2A according to their multicellularity levels as “unicellular” [0], “simple or aggregative multicellularity” [1], or “complex multicellularity” [2]. Simple or aggregative multicellular organisms can switch between living in a group(s) and living as single cells^19^. Complex multicellular organisms have cell differentiation and cannot switch to unicellularity^19,67^. For example, Porifera and Placozoa are non-unicellular and consist of different cell types, therefore we classified them as complex multicellular organisms, and not as “simple or aggregative multicellular” organisms as classified by Aktipis et al.^19^. We also categorized cell-in-cell phenomena from Supplementary Table 2A based on the level of ‘selfishness’ of the interacting cells. The cell-in-cell categories were “no cell-in-cell phenomena reported/found” [0], “heterospecific cell-in-cell phenomena where both cells remain alive” [1], “heterospecific cell-in-cell phenomena where at least one cell dies” [2], “conspecific cell-in-cell phenomena where both cells remain alive” [3], “conspecific cell-in-cell phenomena where at least one cell dies” [4], “conspecific cell-in-cell phenomena where both cells remain alive and at least one of the cells is a neoplastic cell” [5], “conspecific cell-in-cell phenomena where at least one cell dies and at least one of the cells is a neoplastic cell” [6]. We do not assume common origins of cell-in-cell-related phenomena among the taxa within each of the above categories, and thus we cannot assign a single evolutionary time for each category.

### Gene functional information

Within the 115 articles shown in Supplementary Table 2, we searched for any mentioned markers of entosis, cannibalism, phagocytosis, emperitosis, and emperipolesis. We collected the names of the cell-in-cell-related genes and information about their cell-in-cell-related function from these articles.

### Gene age data

We found the human homologs of the cell-in-cell-related genes that we had identified (Supplementary Table 3) and obtained their evolutionary age using a human gene age database published in previous work^68^. These gene ages are determined as the maximum phylogenetic divergence time between humans and the species represented in each gene ontology, as given in the TimeTree database^52,53^. We could not find human homologs of two cell-in-cell-related genes (AlyA and FspA)^24^.

AlyA is a gene found in *Klebsiella pneumoniae*, a gram-negative Enterobacterium. This gene encodes for Alginate lyase, and it is also found in brown (Phaeophyceae) and red algae (Rhodophyta)^69^, which leads to an age of at least 4250 MYA based on the estimated time of divergence^53^.

FspA is a gene found in *Campylobacter jejuni*, also a gram-negative bacterium. It encodes for the Type 3 secretion system protein^70^ and it is found in a great number of eubacteria^71^. This indicates that this gene most likely emerged around 4250 MYA with the evolution of bacteria^53^.

### Supplementary Information

Supplementary Table 1.

Supplementary Tables.

Supplementary Figure 1.

## Data Availability

All data used are shown in the supplementary.
