# Supplementary material for: Cell-in-cell phenomena across the tree of life
Source: Sci Rep. 2024 Mar 29;14:7535. doi: 10.1038/s41598-024-57528-7 (PMC10980697; doi:10.1038/s41598-024-57528-7)
Supplement: Supplementary file 2 — Supplementary Tables. [file 41598_2024_57528_MOESM2_ESM.docx]

# Supplementary Table 2A.

# **Cell-in-cell phenomena across the tree of life.** We obtained data on the levels of multicellularity and cancer from Aktipis et al.^1^ and Fisher et al.^2^. In Archaea, even though an endosymbiosis event involving an Archaeon is widely acknowledged as the event which led to the origin of eukaryotes, there is no evidence of phagocytosis currently happening in Archaea^3^. Similarly, in other taxa, we only present evidence of cell-in-cell phenomena from recently observed events. NA: not available.

| **Taxon (ranking)** | **Level of** | | Cell engulfs **heterospecific** cell & **kills** the engulfed or host cell | Cell engulfs **heterospecific** cell & both cells remain **alive** | Cell engulfs **conspecific** cell & **kills** the engulfed or host cell & both are **non-neoplastic** cells **(includes examples of the host cell not being an immune cell)** | Cell engulfs **conspecific** cell & both cells remain **alive** & both are **non-neoplastic** cells **(includes examples of the host cell not being an immune cell)** | Cell engulfs **conspecific** cell & **kills** the engulfed or host cell & ≥1 is a **neoplastic cell (includes examples of the host cell not being an immune cell)**  **​​** | Cell engulfs **conspecific** cell & both cells remain **alive** & ≥1 is a **neoplastic** **cell (includes examples of the host cell not being an immune cell)** |
| --- | --- | --- | --- | --- | --- | --- | --- | --- |
|  | **Multicellularity** | **Cancer** |  |  |  |  |  |  |
| Vertebrata (subphylum) | Complex multicellularity | Cancer reported | **✓** (phagocytosis)^4–12^ | NA | **✓**  (phagocytosis)^11,13–18^ & (cannibalism)^19,20^ & (entosis)^21–29^ & (efferocytosis)^30^ & (emperipolesis)^31^ & (phagoptosis)^32,33^ & (fusion)^34–36^ | **✓** (emperipolesis)^6,37–39^ & (entosis)^23^ | **✓**^40^ (cannibalism)^41–55^ & (entosis)^27,43–45,56–60^ & (engulfing)^61–65^ & (cell-in-cell)^66–68^ & (internalization)^69^ & (phagocytosis)^70–79^ & (fusion)^80,81^ | **✓** ^40^  (entosis)^43^ & (emperipolesis)^74^ & (cannibalism)^46^ & (internalization)^69^ & (engulfment)^82^ & (cell-in-cell)^68^ |
| Urochordata/Tunicata (subphylum) | Complex multicellularity | Cancer reported | **☓** (no reported phagocytosis)^83^ | **☓** (no reported phagocytosis)^83^ | **✓** (trephocytes within the egg)^84,85^ | NA | NA | NA |
| Cephalochordata (subphylum) | Complex multicellularity | Cancer reported | **✓** (intracellular digestion)^83^ | NA | NA | NA | NA | NA |
| Echinodermata (phylum) | Complex multicellularity | Cancer reported | **✓** (intracellular digestion)^83^ | NA | **✓** (cannibalism)^86^ | **✓** (cannibalism)^86^ | NA | NA |
| Hemichordata (phylum) | Complex multicellularity | No cancer-like phenomena reported | **✓** (intracellular digestion)^83^ | NA | NA | NA | NA | NA |
| Protostomia (unranked) | Complex multicellularity | Cancer reported | **✓** can be dying cells (phagocytosis)^87–90^ | NA | **✓** (phagocytosis)^91–93^ & (fusion of gonad-to-cloaca cells via entosis and then death of the entosed cell)^94^ & (eating nutrient from a dead conspecific)^91^ & (trephocytes within the egg)^84,85^ | NA | NA | NA |
| Cnidaria (phylum) | Complex multicellularity | Cancer reported | **✓** (phagocytosis)^95,96^ | NA | **✓** (nurse cells transfer cytoplasm to oocyte via ring canals, they undergo apoptosis and then phagocytosis)^97^ | NA | NA | NA |
| Placozoa (phylum) | Complex multicellularity | No cancer-like phenomena reported | **✓** (“phagocytosis may be present”)^83^ | **✓** (“phagocytosis may be present”)^83^ | NA | NA | NA | NA |
| Porifera (phylum) | Complex multicellularity | No cancer-like phenomena reported | **✓** (phagocytosis)^96,98–103^ | NA | **✓** (oocytes phagocytose nurse cells)^104,105^ | NA | NA | NA |
| Ctenophora (phylum) | Complex multicellularity | No cancer-like phenomena reported | **✓** (intracellular digestion)^83^ & (phagocytosis)^96^ | NA | NA | NA | NA | NA |
| Choanoflagellata (class) | Simple/aggregative multicellularity | No cancer-like phenomena reported | **✓** (intracellular digestion)^83^ & (phagocytosis)^106^ | NA | NA | NA | NA | NA |
| Ascomycota (division) | Unicellularity, simple or aggregative multicellularity, complex multicellularity | Cancer reported | **☓** (“no phagocytosis has been reported”)^107^ | **☓** (“no phagocytosis has been reported”)^107^ | NA | NA | NA | NA |
| Basidiomycota (division) | Unicellularity, simple or aggregative multicellularity, complex multicellularity | Cancer-like phenomena reported | **☓** (“no phagocytosis has been reported”)^107^ | **☓** (“no phagocytosis has been reported”)^107^ | NA | NA | NA | NA |
| Amoebozoa (phylum) | Unicellularity, simple or aggregative multicellularity | Cancer-like phenomena reported | **✓** (phagocytosis)^108–112^ | **✓** (endosymbiosis)^108^ | NA | NA | NA | NA |
| Embryophyta (clade) | Complex multicellularity | Cancer reported | **✓** whole (endocytosis)^113^ | NA | NA | NA | NA | NA |
| Chlorophyta (division) | Unicellularity, simple or aggregative multicellularity, complex multicellularity | Cancer-like phenomena reported | **✓** whole (bacterivory)^114^ | NA | NA | NA | NA | NA |
| Rhodophyta (division) | Unicellularity, simple or aggregative multicellularity, complex multicellularity | Cancer reported | **☓** (no known phagocytosis)^115^ | **☓** (no known phagocytosis)^115^ | NA | NA | NA | NA |
| Stramenopiles (clade) | Unicellularity, simple or aggregative multicellularity, complex multicellularity | Cancer-like phenomena reported | NA | NA | **✓** (cannibalism)^116^ | NA | NA | NA |
| Archaea (domain) | Unicellularity, simple/aggregative multicellularity | NA | **☓** (no known phagocytosis)^3^ | NA | NA | NA | NA | NA |
| Bacteria (domain) | Unicellularity, simple or aggregative multicellularity | Cancer-like phenomena reported | **✓** (cell-in-cell)^117^ | NA | NA | NA | NA | NA |

# Supplementary Table 2B.

# **Cell-in-cell phenomena in vertebrate species.** All the species below perform heterospecific killing (phagocytosis), but none perform heterospecific cell-in-cell phenomena where both cells remain alive.

| **Species** | Cell engulfs **conspecific** cell & **killing** of the engulfed or host cell & both are **non-neoplastic** cells | Cell engulfs **conspecific** cell & both cells remain **alive** & both are **non-neoplastic** cells | Cell engulfs **conspecific** cell & **killing** of the engulfed or host cell & ≥1 is a **neoplastic cell** | Cell engulfs **conspecific** cell & both cells remain **alive** & ≥1 is a **neoplastic** **cell** |
| --- | --- | --- | --- | --- |
| *Danio rerio* | **✓** (entosis)^22^ | (emperipolesis)^6^ | NA | NA |
| *Sparus aurata* | **✓** (phagocytosis)^8^ | NA | NA | NA |
| *Pelodiscus sinensis* | **✓** (entosis)^21^ | NA | NA | NA |
| *Rattus norvegicus* | **✓** (phagocytosis)^13^ | NA | **✓**^40^ | **✓**^40^ |
| *Mus musculus* | **✓** (phagocytosis)^11,13–15,18^ & (entosis)^23–25^ & (emperipolesis)^31^ & (phagoptosis)^33^ | **✓** (entosis)^23^ | **✓** (engulfment)^61^ | NA |
| *Felis catus* | **✓** (phagocytosis)^118^ | NA | **✓** (cannibalism)^45^ | NA |
| *Canis lupus familiaris* | **✓** (phagocytosis)^12,119^ | NA | **✓** (cannibalism)^45^ | NA |
| *Homo sapiens* | **✓** (cannibalism)^19,20^ & (phagocytosis)^16–18^ & (efferocytosis)^30^ & (entosis)^26–29^ & (engulfment)^16^ & (fusion)^34^ | **✓** (emperipolesis)^37–39^ | **✓** (engulfment)^61–65^ (entosis)^44,56–60^ & (internalization)^69^ & (cannibalism)^41–44,47–55^ & (cell-in-cell)^66–68^ & (phagocytosis)^70–79^ & (fusion)^80,81^ | **✓**(internalization)^69^ & (emperipolesis)^74^ & (engulfment)^82^ & (cell-in-cell)^68^ |

# Supplementary Table 3.

**Genes involved in cell-in-cell phenomena.** The genes are listed in the table from youngest to oldest. We found these cell-in-cell-related genes from our systematic search of the literature. MYA: million years ago. *AlyA and FspA do not have a human homologue (see Methods). In the 6th column we only mention the function of the non-vertebrate genes that have a human homologue. We obtained gene card numbers from Stelzer et al.^120^

| **Markers/regulators of cell-in-cell phenomena (mentioned in reference column)** | **Organism(s) (to which the marker/regulator in the 1st column belongs)** | **Cell-in-cell related function(s) as mentioned in the reference(s)** | **Reference** | **Human homologue** | **Cell-in-cell related function(s) of the human homologue** | **Gene card number of the human homologue** | **Age of the human homologue gene (MYA)** |
| --- | --- | --- | --- | --- | --- | --- | --- |
| ICAM-1 | humans | involved in forming heterotypic cell-in-cell structures | ^68^ | ICAM1 | – | GC19P010749 | 361.8 |
| LFA-2 | humans | involved in forming heterotypic cell-in-cell structures | ^68^ | CD2 | – | GC01P116754 | 400.1 |
| lysozyme | humans | cannibalistic marker | ^121^ | LYZ | – | GC12P069348 | 762.8 |
| CD62L | humans | involved in forming heterotypic cell-in-cell structures | ^68^ | SELL | – | GC01M169690 | 891.7 |
| E-cadherin | humans, cats, dogs | marker of cell-in-cell structures, including cannibalizing and entotic cells; required for homotypic cell-in-cell structure formation; forms concentrated foci at contact points between cells and is required for entosis; required for the formation of adhesive junctions between cells during entosis | ^23,27,29,43,45,56,59,61,68,69,82,122,123^ | CDH1 | – | GC16P068737 | 937.4 |
| N-cadherin | humans | aids in the formation of cell contacts required for homotypic cell-in-cell formation; a cell-cell adhesion molecule that facilitates cell-in-cell invasion | ^57^ | CDH2 | – | GC18M028088 | 937.4 |
| P-cadherin | humans | required for cell-cell adhesion and cell uptake during entosis | ^43,123^ | CDH3 | – | GC16P068637 | 937.4 |
| F4/80 | humans | macrophage and phagocyte marker | ^24,51^ | ADGRE1 | – | GC19P006887 | 940 |
| Crq/Dsb | *Drosophila* | related to phagosome maturation | ^17^ | CD36 | mediates phagocytosis (e.g., phagocytosis of *Plasmodium falciparum*)^124^ | GC07P080369 | 940 |
| CD68 | humans | phagocytic, macrophage, and cannibalistic marker expressed by cannibal and internalized cells | ^51,52,54,63,71,121,125^ | CD68 | – | GC17P007579 | 940 |
| a-catenin | humans | core adhesive component indispensable for entosis | ^44^ | CTNNA2 | – | GC02P079185 | 940 |
| β-catenin | humans | required for cell–cell adhesion; required for cell internalization during homotypic cell-in-cell phenomena; required for forming adhesive junctions and is gradually degraded during entosis | ^29,59,68,82^ | CTNNB1 | – | GC03P041194 | 940 |
| ezrin | humans | a cell cortical protein involved in forming heterotypic cell-in-cell structures, including cannibalism | ^68,123^ | EZR | – | GC06M158765 | 940 |
| LAMP, LAMP1 | *Drosophila*, turtles, humans | phagolysosome marker expressed by epidermal cells | ^17,26^ | LAMP1 | – | GC13P113297 | 940 |
| actomyosin | humans | required for cell-cell adhesion prior to ingesting neighboring live cells | ^126^ | MYH1 | – | GC17M010492 | 940 |
| myosin | zebrafish | drives the initial steps of entosis | ^22,126,127^ | MYH2 | – | GC17M010521 | 940 |
| p53 | humans | mutant p53 increases cellular engulfment leading to cell-in-cell phenomena | ^82^ | TP53 | – | GC17M007661 | 940 |
| WASH | *Dictyostelium* | involved in vesicular trafficking and phagosome maturation | ^112^ | WASF1 | cell-in-cell related (e.g., “receptor-mediated endocytosis”) | GC06M110099 | 1184.5 |
| DIA1 | humans | regulates cell tension | ^123^ | DIAPH1 | – | GC05M141516 | 1215.8 |
| Rho, RhoA | metazoa | regulates actin polymerization required for cell-cell adhesion, contractility of the internalized cell, phagocytosis, and entosis | ^22,62,82,92,107,126,128,129^ | RHOA | – | GC03M049359 | 1215.8 |
| cathepsin G | mice | regulates the ability of neutrophils to kill *Staphylococcus aureus* pathogens | ^112^ | CTSG | – | GC14M024573 | 1362 |
| CD163 | humans | marker of macrophages involved in cell-in-cell phenomena | ^63^ | CD163 | – | GC12M008027 | 1369 |
| CD204 | humans | "M2-polarization macrophage marker” | ^80^ | MSR1 | – | GC08M016107 | 1369 |
| K-Ras | humans | molecular marker of entosis | ^82^ | KRAS | – | GC12M025204 | 1381.2 |
| Nox2 | mammals | involved in intracellular killing of bacteria | ^112^ | CYBB | – | GC0XP037780 | 1530.3 |
| TM9SF4, Phg1a | humans, *Dictyostelium* | associated with tumor cell cannibalism; controls “intracellular transport and stability of membrane proteins" and intracellular killing | ^112,130^ | TM9SF4 | – | GC20P032109 | 1530.3 |
| actin | zebrafish | required for cell-cell adhesion prior to the ingestion of neighboring live cells | ^6,126^ | ACTB | – | GC07M005527 | 2269.5 |
| Cdc42 | bacteria, humans | regulates actin polymerization and phagocytosis; required for entosis | ^22,44,107,128^ | CDC42 | – | GC01P022052 | 2269.5 |
| LvsB | *Dictyostelium* | involved in lysosome maturation | ^112^ | LYST | regulates phagosome maturation and lysosome organization^131^ | GC01M235661 | 2269.5 |
| AMPK | humans | regulates and is required for entosis; glucose “suppresses entosis induction by inhibiting activity of the energy-sensing AMP-activated protein kinase (AMPK)”; cells “with the lowest energy levels, and concomitantly the highest levels of AMPK activity, are sacrificed to feed those with lowered AMPK activity" | ^123^ | PRKAA1 | – | GC05M040759 | 2269.5 |
|  |  |  |  | PRKAB1 |  | GC12P119632 |  |
|  |  |  |  | PRKAB2 |  | GC01M147155 |  |
|  |  |  |  | PRKAG1 |  | GC12M049002 |  |
|  |  |  |  | PRKAG2 |  | GC07M151556 |  |
|  |  |  |  | PRKAG3 |  | GC02M218823 |  |
| Rab7a | *Drosophila*, humans | phagocytic and late phagosome/phagolysosome marker | ^17,71^ | RAB7A | – | GC03P134973 | 2269.5 |
| FAT1 | humans | mediates cell-cell adhesion | ^19^ | FAT1 | – | GC04M186587 | 2535.8 |
| NRAMP1 | *Dictyostelium*, mammals | a metal ion transporter on the phagosomal membrane of mammalian macrophages involved in intracellular bacterial killing | ^112^ | SLC11A1 | – | GC02P218382 | 3556.3 |
| AlyA | *Dictyostelium* | responsible for almost 50% of the total cellular lysozyme activity | ^112^ | * |  |  | 4250 |
| FspA | *Dictyostelium* | may act as a receptor or regulator in the folate-sensing pathway | ^112^ | * |  |  | 4250 |

# Supplementary Table 4.

**Examples of conspecific cell-in-cell behaviors (A), cannibalistic multicellular animal behaviors (B), and the direct fitness benefit to the host, prey, or virus.** We have placed examples of A and B that are conceptually similar in the same row but in different columns (separated by a vertical black line).

| **Event** | **Direct fitness benefit to** | | | **Reference** | **Event** | **Direct fitness benefit to** | | | **Reference** |
| --- | --- | --- | --- | --- | --- | --- | --- | --- | --- |
|  | **predator** | **prey** | **virus/prion within prey** |  |  | **predator** | **prey** | **virus/prion within prey** |  |
| **A** |  |  |  |  | **B** |  |  |  |  |
| examples of cell cannibalism in Vertebrata in Table 1 |  |  |  |  | cannibalism has happened in 16 families of spiders, fall armyworms (*Spodoptera frugiperda*), the American alligator (*Alligator mississippiensis*), 13 avian families, 75 species of mammals [including hedgehogs, voles, mice, woodrats, rabbits, shrews, moles, and 14 species of carnivores such as polar bears, pumas (*Puma concolor*), lynx (*Lynx lynx*), leopards (*Panthera pardus*), and sea lions (*Phocarctos hookeri*)] | + |  |  | ^132,133^ |
| under hypoxia, acidity, mitosis^44^, and starvation (termed “the great equalizer”^132^, i.e., regardless of relatedness, both kin and non-kin are eaten, expressed as “Ι’m starving, I’ll eat anything!”, and “No one can… love his neighbors on an empty stomach." - Woodrow Wilson)/hibernation/absence of vasculature in a multicellular body, a cell that eats a related cell has a resource and survival benefit in comparison to a non-cannibalistic cell | + |  |  | ^21,41,50,134,135^ | during food scarcity, eusocial species, such as honeybees (*Apis mellifera*), cannibalize their newborn | + |  |  | ^136^ |
|  |  |  |  |  | during food scarcity, voles, mice, woodrats, rabbits, shrews, moles, and hedgehogs cannibalize their young | + |  |  | ^132^ |
|  |  |  |  |  | cannibalism between mantises that have been starved | + |  |  | ^132^ |
|  |  |  |  |  | sibling cannibalism between acorn woodpeckers due to asynchronous hatching | + |  |  | ^132^ |
|  |  |  |  |  | sibling cannibalism between snowy egret (*Leucophoyx thula*) hatchlings due to unequal provision of hormones to the eggs | + |  |  | ^132^ |
| the resulting possible change in the shape of the host cell after the cell-in-cell event^137^ may also help it adapt in the new microenvironment(s) during metastasis or development^138^, e.g., uterine luminal epithelial cells enter blastocyst trophoblast cells during the first few days of pregnancy | + |  |  | ^24^ | cannibalism can be advantageous when flour beetles (*Tribolium castaneum*) colonize new environments | + |  |  | ^139^ |
| the engulfed cell can also become the host cell’s ‘passport’, or ‘passe-partout’, entitled “I am what I eat”. In other words, “having a local inside me helps me pass the border”. For instance a cancer cell fusing with a macrophage and via horizontal gene transfer gaining genetic ‘knowledge’ about a different environment, helping the cancer cell avoid the immune system, become more radioresistant, express the macrophage-specific marker CD163, and become more adapted and ‘blended’/‘accepted’ in the new environment(s) and thus be more metastatic | + |  |  | ^140–145^ |  |  |  |  |  |
| a slime mold cell with the ability to habituate towards a repellent can transfer this trait to the host cell that ate it | + |  |  | ^146^ | humans drank other people’s blood in the 18th century as medicine | + |  |  | ^132^ |
| similar to a Trojan horse, the engulfed cell (e.g., lymphocyte) can become more toxic inside the host cell (e.g., neoplastic cell) and kill the host cell while the engulfed cell escapes |  | + |  | ^147–149^ |  |  |  |  |  |
| multiple myoblast cells fuse together leading to the formation of multinucleated myoblast cells later in development | + |  |  | ^35^ | adelphophagy or sibling cannibalism between raptors and sand tiger shark embryos (*Carcharias taurus*) | + |  |  | ^132^ |
|  |  |  |  |  | inability to recognise conspecifics early in development (in cases where kin recognition mechanisms have not yet developed), starvation and lack of other food sources, and overcrowding can lead to cannibalism in clams, insects, and scorpions | + |  |  | ^132^ |
| a non-digested engulfed cell inside a dormant neoplastic cell is protected from possible attack by other external biological/chemical agents |  | + |  | ^65^ | mouthbrooding in cichlids results in some newborn being eaten | + | + |  | ^132^ |
| a cancer cell that eats immune cells, which are about to eat the cancer cell, has a survival benefit | + |  |  | ^41,73^ | the Aztecs, Caribs, Lendi, Batak, Dyak, Sawney Bean’s clan, Iroquois, Nuuchahnulth, Ancestral Puebloans, Tupinambá, Wari’, Marquesans, Mianmin, Asmat, Fijians, and Maori have practiced exocannibalism, i.e., eating humans that are their enemies | + |  |  | ^150^ |
| a prey cell enters a host cell and commits apoptosis | + |  |  | ^151^ |  |  |  |  |  |
| the engulfed genetically related cell may benefit from the predation event if its exact genome is passed to its host’s (relative’s) nucleus and on to the next generation at every host cell division |  | + |  | ^152–154^ | monkeys, rodents, lagomorphs, carnivores, primates, and most artiodactyls eat their placenta after giving birth | + |  |  | ^132^ |
| Epstein-Barr virus can infect another cell when its host cell is eaten by another cell |  |  | + | ^155^ | cannibalism in humans has happened during famine in ancient Egypt, ancient Greece, ancient Rome, Persia, India, China, Japan, between 793 CE and 1317 CE in Europe, and in the 1950s in Papua New Guinea (which led to the spread of abnormally folded proteins, called prions, causing the fatal disease kuru) | + |  | + | ^132^ |

# Supplementary References

1 Aktipis CA, Boddy AM, Jansen G, Hibner U, Hochberg ME, Maley CC *et al.* Cancer across the tree of life: cooperation and cheating in multicellularity. *Philos Trans R Soc Lond B Biol Sci* 2015; **370**: 20140219–20140219.

2 Fisher RM, Cornwallis CK, West SA. Group Formation, Relatedness, and the Evolution of Multicellularity. *Curr Biol* 2013; **23**: 1120–1125.

3 Martin WF, Tielens AGM, Mentel M, Garg SG, Gould SB. The Physiology of Phagocytosis in the Context of Mitochondrial Origin. *Microbiol Mol Biol Rev* 2017; **81**. doi:10.1128/MMBR.00008-17.

4 Masud S, Prajsnar TK, Torraca V, Lamers GEM, Benning M, Van Der Vaart M *et al.* Macrophages target Salmonella by Lc3-associated phagocytosis in a systemic infection model. *Autophagy* 2019; **15**: 796–812.

5 Rasmussen JP, Sack GS, Martin SM, Sagasti A. Vertebrate epidermal cells are broad-specificity phagocytes that clear sensory axon debris. *J Neurosci* 2015; **35**: 559–570.

6 Chia JSM, Wall ES, Wee CL, Rowland TAJ, Cheng R-K, Cheow K *et al.* Bacteria evoke alarm behaviour in zebrafish. *Nat Commun* 2019; **10**: 3831.

7 Ainsworth AJ. Carbohydrate and lectin interactions with Edwardsiella ictaluri and channel catfish, Ictalurus punctatus (Rafinesque), anterior kidney leucocytes and hepatocytes. *J Fish Dis* 1993; **16**: 449–459.

8 Rodríguez A, Esteban MA, Meseguer J. A mannose-receptor is possibly involved in the phagocytosis of Saccharomyces cerevisiae by seabream (Sparus aurata L.) leucocytes. *Fish Shellfish Immunol* 2003; **14**: 375–388.

9 Li Q, Cheng H, Liu Y, Wang X, He F, Tang L. Activation of mTORC1 by LSECtin in macrophages directs intestinal repair in inflammatory bowel disease. *Cell Death Dis* 2020; **11**: 918.

10 Rogers CL. *Identifying GSDME/DFNA5 as a Multifaceted Regulator of the Apoptotic Program*. 2019.http://login.ezproxy1.lib.asu.edu/login?url=https://www.proquest.com/dissertations-theses/identifying-gsdme-dfna5-as-multifaceted-regulator/docview/2228979100/se-2.

11 Bonilla DL, Bhattacharya A, Sha Y, Xu Y, Xiang Q, Kan A *et al.* Autophagy regulates phagocytosis by modulating the expression of scavenger receptors. *Immunity* 2013; **39**: 537–547.

12 Wagner A, Junginger J, Lemensieck F, Hewicker-Trautwein M. Immunohistochemical characterization of gastrointestinal macrophages/phagocytes in dogs with inflammatory bowel disease (IBD) and non-IBD dogs. *Vet Immunol Immunopathol* 2018; **197**: 49–57.

13 Kelp NC. *Progesterone Receptor Membrane Components 1 and 2 in Female Reproductive Physiology and Pathology*. 2017.http://login.ezproxy1.lib.asu.edu/login?url=https://www.proquest.com/dissertations-theses/progesterone-receptor-membrane-components-1-2/docview/1987568440/se-2.

14 Rőszer, Menéndez-Gutiérrez. Autoimmune kidney disease and impaired engulfment of apoptotic cells in mice with macrophage peroxisome proliferator-activated receptor γ or retinoid X receptor α …. *The Journal of* 2011.https://www.jimmunol.org/content/186/1/621.short.

15 Monks J, Smith-Steinhart C, Kruk ER, Fadok VA, Henson PM. Epithelial cells remove apoptotic epithelial cells during post-lactation involution of the mouse mammary gland. *Biol Reprod* 2008; **78**: 586–594.

16 Kevany BM, Palczewski K. Phagocytosis of retinal rod and cone photoreceptors. *Physiology*  2010; **25**: 8–15.

17 Han C, Song Y, Xiao H, Wang D, Franc NC, Jan LY *et al.* Epidermal cells are the primary phagocytes in the fragmentation and clearance of degenerating dendrites in Drosophila. *Neuron* 2014; **81**: 544–560.

18 Fletcher K, Ulferts R, Jacquin E, Veith T, Gammoh N, Arasteh JM *et al.* The WD 40 domain of ATG 16L1 is required for its non‐canonical role in lipidation of LC 3 at single membranes. *EMBO J* 2018; **37**: e97840.

19 Bauer MF, Hader M, Hecht M, Büttner-Herold M, Fietkau R, Distel LVR. Cell-in-cell phenomenon: leukocyte engulfment by non-tumorigenic cells and cancer cell lines. *BMC Mol Cell Biol* 2021; **22**: 39.

20 Kalele KP, Patil KP, Nayyar AS, Sasane RS. Atypical Lymphocytes and Cellular Cannibalism: A Phenomenon, First of its Kind to be Discovered in Chronic Periapical Lesions. *J Clin Diagn Res* 2016; **10**: ZC01-4.

21 Ahmed N, Yang P, Huang Y, Chen H, Liu T, Wang L *et al.* Entosis Acts as a Novel Way within Sertoli Cells to Eliminate Spermatozoa in Seminiferous Tubule. *Front Physiol* 2017; **8**: 361.

22 Armistead J, Hatzold J, van Roye A, Fahle E, Hammerschmidt M. Entosis and apical cell extrusion constitute a tumor-suppressive mechanism downstream of Matriptase. *J Cell Biol* 2020; **219**. doi:10.1083/jcb.201905190.

23 Sottile F, Aulicino F, Theka I, Cosma MP. Mesenchymal stem cells generate distinct functional hybrids in vitro via cell fusion or entosis. *Sci Rep* 2016; **6**: 36863.

24 Li Y, Sun X, Dey SK. Entosis allows timely elimination of the luminal epithelial barrier for embryo implantation. *Cell Rep* 2015; **11**: 358–365.

25 Segawa K, Yanagihashi Y, Yamada K, Suzuki C, Uchiyama Y, Nagata S. Phospholipid flippases enable precursor B cells to flee engulfment by macrophages. *Proc Natl Acad Sci U S A* 2018; **115**: 12212–12217.

26 Florey O, Kim SE, Sandoval CP, Haynes CM, Overholtzer M. Autophagy machinery mediates macroendocytic processing and entotic cell death by targeting single membranes. *Nat Cell Biol* 2011; **13**: 1335–1343.

27 Krajcovic M, Johnson NB, Sun Q, Normand G, Hoover N, Yao E *et al.* A non-genetic route to aneuploidy in human cancers. *Nat Cell Biol* 2011; **13**: 324–330.

28 Khalkhali-Ellis Z, Goossens W, Margaryan NV, Hendrix MJC. Cleavage of Histone 3 by Cathepsin D in the involuting mammary gland. *PLoS One* 2014; **9**: e103230.

29 Garanina AS, Khashba LA, Onishchenko GE. Stages of cell cannibalism--entosis--in normal human keratinocyte culture. *Biochemistry*  2015; **80**: 1469–1477.

30 Caruso RA, Fedele F, Di Bella C, Mazzon E, Rigoli L. Foveolar cells phagocytose apoptotic neutrophils in chronic active Helicobacter pylori gastritis. *Virchows Arch* 2012; **461**: 489–494.

31 Benseler V, Warren A, Vo M, Holz LE, Tay SS, Le Couteur DG *et al.* Hepatocyte entry leads to degradation of autoreactive CD8 T cells. *Proc Natl Acad Sci U S A* 2011; **108**: 16735–16740.

32 Neher JJ, Neniskyte U, Brown GC. Primary phagocytosis of neurons by inflamed microglia: potential roles in neurodegeneration. *Front Pharmacol* 2012; **3**: 27.

33 Hornik TC, Vilalta A, Brown GC. Activated microglia cause reversible apoptosis of pheochromocytoma cells, inducing their cell death by phagocytosis. *J Cell Sci* 2016; **129**: 65–79.

34 Melzer C, von der Ohe J, Hass R. In Vitro Fusion of Normal and Neoplastic Breast Epithelial Cells with Human Mesenchymal Stroma/Stem Cells Partially Involves Tumor Necrosis Factor Receptor Signaling. *Stem Cells* 2018; **36**: 977–989.

35 Kim JH, Jin P, Duan R, Chen EH. Mechanisms of myoblast fusion during muscle development. *Curr Opin Genet Dev* 2015; **32**: 162–170.

36 Lizier M, Castelli A, Montagna C, Lucchini F, Vezzoni P, Faggioli F. Cell fusion in the liver, revisited. *World J Hepatol* 2018; **10**: 213–221.

37 Tefferi A. Pathogenesis of myelofibrosis with myeloid metaplasia. *J Clin Oncol* 2005; **23**: 8520–8530.

38 Schmitt A, Drouin A, Massé J-M, Guichard J, Shagraoui H, Cramer EM. Polymorphonuclear neutrophil and megakaryocyte mutual involvement in myelofibrosis pathogenesis. *Leuk Lymphoma* 2002; **43**: 719–724.

39 Schmitt A, Jouault H, Guichard J, Wendling F, Drouin A, Cramer EM. Pathologic interaction between megakaryocytes and polymorphonuclear leukocytes in myelofibrosis. *Blood* 2000; **96**: 1342–1347.

40 Logothetou-Rella H. Glycosaminoglycan-sac formation in vitro. Interactions between normal and malignant cells. *Histol Histopathol* 1994; **9**: 243–249.

41 Lugini L, Matarrese P, Tinari A, Lozupone F, Federici C, Iessi E *et al.* Cannibalism of live lymphocytes by human metastatic but not primary melanoma cells. *Cancer Res* 2006; **66**: 3629–3638.

42 Fais S. Cannibalism: a way to feed on metastatic tumors. *Cancer Lett* 2007; **258**: 155–164.

43 Overholtzer M, Mailleux AA, Mouneimne G, Normand G, Schnitt SJ, King RW *et al.* A nonapoptotic cell death process, entosis, that occurs by cell-in-cell invasion. *Cell* 2007; **131**: 966–979.

44 Durgan J, Tseng Y-Y, Hamann JC, Domart M-C, Collinson L, Hall A *et al.* Mitosis can drive cell cannibalism through entosis. *Elife* 2017; **6**. doi:10.7554/eLife.27134.

45 Ferreira FC, Soares MJ, Carvalho S, Borralho L, Vicente G, Branco S *et al.* Four cases of cell cannibalism in highly malignant feline and canine tumors. *Diagn Pathol* 2015; **10**: 199.

46 Sharma N, Dey P. Cell cannibalism and cancer. *Diagn Cytopathol* 2011; **39**: 229–233.

47 Conner CM. *To the Edge of Apoptotic Cell Death and Back*. 2021.http://login.ezproxy1.lib.asu.edu/login?url=https://www.proquest.com/dissertations-theses/edge-apoptotic-cell-death-back/docview/2531366053/se-2.

48 Brouwer, Ley, Feltkamp, Elema. Serum-dependent “cannibalism” and autodestruction in cultures of human small cell carcinoma of the lung. *Cancer Res* 1984.https://aacrjournals.org/cancerres/article-abstract/44/7/2947/488619.

49 Sarode GS, Sarode SC, Karmarkar S. Complex cannibalism: an unusual finding in oral squamous cell carcinoma. *Oral Oncol* 2012; **48**: e4-6.

50 Arya P, Khalbuss WE, Monaco SE, Pantanowitz L. Salivary duct carcinoma with striking neutrophil-tumor cell cannibalism. *Cytojournal* 2011; **8**: 15.

51 Cano CE, Sandí MJ, Hamidi T, Calvo EL, Turrini O, Bartholin L *et al.* Homotypic cell cannibalism, a cell-death process regulated by the nuclear protein 1, opposes to metastasis in pancreatic cancer. *EMBO Mol Med* 2012; **4**: 964–979.

52 Fernandez-Flores A. Cannibalism in a benign soft tissue tumor (giant-cell tumor of the tendon sheath, localized type): a study of 66 cases. *Rom J Morphol Embryol* 2012; **53**: 15–22.

53 Caruso RA, Rigoli L, Parisi A, Fedele F, Bonanno A, Paparo D *et al.* Neutrophil-rich gastric carcinomas: light and electron microscopic study of 9 cases with particular reference to neutrophil apoptosis. *Ultrastruct Pathol* 2013; **37**: 164–170.

54 Siddiqui S, Singh A, Faizi N, Khalid A. Cell cannibalism in oral cancer: A sign of aggressiveness, de-evolution, and retroversion of multicellularity. *J Cancer Res Ther* 2019; **15**: 631–637.

55 Lozupone F, Perdicchio M, Brambilla D, Borghi M, Meschini S, Barca S *et al.* The human homologue of Dictyostelium discoideum phg1A is expressed by human metastatic melanoma cells. *EMBO Rep* 2009; **10**: 1348–1354.

56 Sun Q, Luo T, Ren Y, Florey O, Shirasawa S, Sasazuki T *et al.* Competition between human cells by entosis. *Cell Res* 2014; **24**: 1299–1310.

57 Khalkar P, Díaz-Argelich N, Antonio Palop J, Sanmartín C, Fernandes AP. Novel Methylselenoesters Induce Programed Cell Death via Entosis in Pancreatic Cancer Cells. *Int J Mol Sci* 2018; **19**. doi:10.3390/ijms19102849.

58 Lai Y, Lim D, Tan P-H, Leung TK-C, Yip GW-C, Bay B-H. Silencing the Metallothionein-2A gene induces entosis in adherent MCF-7 breast cancer cells. *Anat Rec*  2010; **293**: 1685–1691.

59 Kisurina-Evgenieva OP, Khashba LA, Mamichev IA, Savitskaya MA, Onishchenko GE. Entosis and cell cycle in tumor cell culture. *Cell tissue biol* 2019; **13**: 8–17.

60 Balvan J, Gumulec J, Raudenska M, Krizova A, Stepka P, Babula P *et al.* Oxidative Stress Resistance in Metastatic Prostate Cancer: Renewal by Self-Eating. *PLoS One* 2015; **10**: e0145016.

61 Tonnessen-Murray CA, Frey WD, Rao SG, Shahbandi A, Ungerleider NA, Olayiwola JO *et al.* Chemotherapy-induced senescent cancer cells engulf other cells to enhance their survival. *J Cell Biol* 2019; **218**: 3827–3844.

62 Abreu M, Sealy L. Cells expressing the C/EBPbeta isoform, LIP, engulf their neighbors. 2012.https://journals.plos.org/plosone/article?id=10.1371/journal.pone.0041807.

63 Huang H, Chen A, Wang T, Wang M, Ning X, He M *et al.* Detecting cell-in-cell structures in human tumor samples by E-cadherin/CD68/CD45 triple staining. *Oncotarget* 2015; **6**: 20278–20287.

64 Wang S, He M, Li L, Liang Z, Zou Z, Tao A. Cell-in-Cell Death Is Not Restricted by Caspase-3 Deficiency in MCF-7 Cells. *J Breast Cancer* 2016; **19**: 231–241.

65 Bartosh TJ, Ullah M, Zeitouni S, Beaver J, Prockop DJ. Cancer cells enter dormancy after cannibalizing mesenchymal stem/stromal cells (MSCs). *Proc Natl Acad Sci U S A* 2016; **113**: E6447–E6456.

66 Chao K-C, Yang H-T, Chen M-W. Human umbilical cord mesenchymal stem cells suppress breast cancer tumourigenesis through direct cell-cell contact and internalization. *J Cell Mol Med* 2012; **16**: 1803–1815.

67 Ma Y, Hao X, Zhang S, Zhang J. The in vitro and in vivo effects of human umbilical cord mesenchymal stem cells on the growth of breast cancer cells. *Breast Cancer Res Treat* 2012; **133**: 473–485.

68 Chen Y-H, Wang S, He M-F, Wang Y, Zhao H, Zhu H-Y *et al.* Prevalence of heterotypic tumor/immune cell-in-cell structure in vitro and in vivo leading to formation of aneuploidy. *PLoS One* 2013; **8**: e59418.

69 Wang S, Guo Z, Xia P, Liu T, Wang J, Li S *et al.* Internalization of NK cells into tumor cells requires ezrin and leads to programmed cell-in-cell death. *Cell Res* 2009; **19**: 1350–1362.

70 DeSimone PA, East R, Powell RD Jr. Phagocytic tumor cell activity in oat cell carcinoma of the lung. *Hum Pathol* 1980; **11**: 535–539.

71 Lugini L, Lozupone F, Matarrese P, Funaro C, Luciani F, Malorni W *et al.* Potent phagocytic activity discriminates metastatic and primary human malignant melanomas: a key role of ezrin. *Lab Invest* 2003; **83**: 1555–1567.

72 Silverman JF, Dabbs DJ, Finley JL, Geisinger KR. Fine-needle aspiration biopsy of pleomorphic (giant cell) carcinoma of the pancreas. Cytologic, immunocytochemical, and ultrastructural findings. *Am J Clin Pathol* 1988; **89**: 714–720.

73 Caruso RA, Muda AO, Bersiga A, Rigoli L, Inferrera C. Morphological evidence of neutrophil-tumor cell phagocytosis (cannibalism) in human gastric adenocarcinomas. *Ultrastruct Pathol* 2002; **26**: 315–321.

74 Singhal N, Handa U, Bansal C, Mohan H. Neutrophil phagocytosis by tumor cells--a cytological study. *Diagn Cytopathol* 2011; **39**: 553–555.

75 Bansal C, Tiwari V, Singh U, Srivastava A, Misra J. Cell Cannibalism: A cytological study in effusion samples. *J Cytol* 2011; **28**: 57–60.

76 Onuma H, Komatsu T, Arita M, Hanaoka K, Ueno T, Terai T *et al.* Rapidly rendering cells phagocytic through a cell surface display technique and concurrent Rac activation. *Sci Signal* 2014; **7**: rs4.

77 Barresi V, Branca G, Ieni A, Rigoli L, Tuccari G, Caruso RA. Phagocytosis (cannibalism) of apoptotic neutrophils by tumor cells in gastric micropapillary carcinomas. *World J Gastroenterol* 2015; **21**: 5548–5554.

78 Gilloteaux J, Ruffo C, Jamison JM, Summers JL. Modes of internalizations of human prostate carcinoma (DU145) cells in vitro and in murine xenotransplants. *Ultrastruct Pathol* 2016; **40**: 231–239.

79 Velmurugan R, Ramakrishnan S, Kim M, Ober RJ, Ward ES. Phagocytosis of antibody-opsonized tumor cells leads to the formation of a discrete vacuolar compartment in macrophages. *Traffic* 2018; **19**: 273–284.

80 Clawson GA, Matters GL, Xin P, McGovern C, Wafula E, dePamphilis C *et al.* “Stealth dissemination” of macrophage-tumor cell fusions cultured from blood of patients with pancreatic ductal adenocarcinoma. *PLoS One* 2017; **12**: e0184451.

81 Oliveira MN, Pillat MM, Motaln H, Ulrich H, Lah TT. Kinin-B1 Receptor Stimulation Promotes Invasion and is Involved in Cell-Cell Interaction of Co-Cultured Glioblastoma and Mesenchymal Stem Cells. *Sci Rep* 2018; **8**: 1299.

82 Mackay HL, Moore D, Hall C, Birkbak NJ, Jamal-Hanjani M, Karim SA *et al.* Genomic instability in mutant p53 cancer cells upon entotic engulfment. *Nat Commun* 2018; **9**: 3070.

83 Steinmetz PRH. A non-bilaterian perspective on the development and evolution of animal digestive systems. *Cell Tissue Res* 2019; **377**: 321–339.

84 Liebman. On trephoeytes and trephocytosis; a study on the role of leucocytes in nutrition and growth. *Growth* 1946.https://www.cabdirect.org/cabdirect/abstract/19461404589.

85 Liebman E. The trephocytes and their functions. *Experientia* 1947; **3**: 442–451.

86 Liebman E. The leucocytes of Arbacia punctulata. *Biol Bull* 1950; **98**: 46–59.

87 Mondragon AA. *Investigation of Non-Autonomous Control of Cell Death and Corpse Clearance in the Ovary of Drosophila melanogaster*. 2019.http://login.ezproxy1.lib.asu.edu/login?url=https://www.proquest.com/dissertations-theses/investigation-non-autonomous-control-cell-death/docview/2196977282/se-2.

88 Hoeppner DJ, Hengartner MO, Schnabel R. Engulfment genes cooperate with ced-3 to promote cell death in Caenorhabditis elegans. *Nature* 2001; **412**: 202–206.

89 Reddien PW, Cameron S, Horvitz HR. Phagocytosis promotes programmed cell death in C. elegans. *Nature* 2001; **412**: 198–202.

90 Johnsen HL, Horvitz HR. Both the apoptotic suicide pathway and phagocytosis are required for a programmed cell death in Caenorhabditis elegans. *BMC Biol* 2016; **14**: 39.

91 Abdullayev I. *Global Regulation of Gene Expression in Stem Cells and Regeneration*. 2017.https://openarchive.ki.se/xmlui/handle/10616/45994.

92 Hurwitz ME, Vanderzalm PJ, Bloom L, Goldman J, Garriga G, Robert Horvitz H. Abl Kinase Inhibits the Engulfment of Apopotic Cells in Caenorhabditis elegans. *PLoS Biol* 2009; **7**: e1000099.

93 Poels J, Spasić MR, Gistelinck M, Mutert J, Schellens A, Callaerts P *et al.* Autophagy and phagocytosis-like cell cannibalism exert opposing effects on cellular survival during metabolic stress. *Cell Death Differ* 2012; **19**: 1590–1601.

94 Lee Y, Hamann JC, Pellegrino M, Durgan J, Domart M-C, Collinson LM *et al.* Entosis Controls a Developmental Cell Clearance in C. elegans. *Cell Rep* 2019; **26**: 3212-3220.e4.

95 Metschnikoff II. Eine neue Entzündungstheorie. *Allg Wiener Med Ztg* 1884; **29**: 307–332.

96 Lancaster CE, Ho CY, Hipolito VEB, Botelho RJ, Terebiznik MR. Phagocytosis: what’s on the menu? Biochemistry and Cell Biology. 2019; **97**: 21–29.

97 Alexandrova O, Schade M, Böttger A, David CN. Oogenesis in Hydra: nurse cells transfer cytoplasm directly to the growing oocyte. *Dev Biol* 2005; **281**: 91–101.

98 Müller WEG, Müller IM. Origin of the metazoan immune system: identification of the molecules and their functions in sponges. *Integr Comp Biol* 2003; **43**: 281–292.

99 Wehrl M, Steinert M, Hentschel U. Bacterial uptake by the marine sponge Aplysina aerophoba. *Microb Ecol* 2007; **53**: 355–365.

100 Vacelet J, Duport E. Prey capture and digestion in the carnivorous sponge Asbestopluma hypogea (Porifera: Demospongiae). *Zoomorphology* 2004; **123**: 179–190.

101 Mukherjee S, Ray M, Ray S. Phagocytic efficiency and cytotoxic responses of Indian freshwater sponge (Eunapius carteri) cells isolated by density gradient centrifugation and flow cytometry: a morphofunctional analysis. *Zoology*  2015; **118**: 8–18.

102 Barreda DR, Neely HR, Flajnik MF. Evolution of Myeloid Cells. *Microbiol Spectr* 2016; **4**. doi:10.1128/microbiolspec.MCHD-0007-2015.

103 Leys SP, Eerkes-Medrano DI. Feeding in a calcareous sponge: particle uptake by pseudopodia. *Biol Bull* 2006; **211**: 157–171.

104 Fell PE. The involvement of nurse cells in oogenesis and embryonic development in the marine sponge,Haliclona ecbasis. *J Morphol* 1969; **127**: 133–149.

105 Schmitt S, Angermeier H, Schiller R, Lindquist N, Hentschel U. Molecular microbial diversity survey of sponge reproductive stages and mechanistic insights into vertical transmission of microbial symbionts. *Appl Environ Microbiol* 2008; **74**: 7694–7708.

106 Dayel MJ, King N. Prey capture and phagocytosis in the choanoflagellate Salpingoeca rosetta. *PLoS One* 2014; **9**: e95577.

107 Yutin N, Wolf MY, Wolf YI, Koonin EV. The origins of phagocytosis and eukaryogenesis. *Biol Direct* 2009; **4**: 9.

108 Jeon KW. Bacterial endosymbiosis in amoebae. *Trends Cell Biol* 1995; **5**: 137–140.

109 Cornillon S, Pech E, Benghezal M, Ravanel K, Gaynor E, Letourneur F *et al.* Phg1p is a nine-transmembrane protein superfamily member involved in dictyostelium adhesion and phagocytosis. *J Biol Chem* 2000; **275**: 34287–34292.

110 Benghezal M, Fauvarque M-O, Tournebize R, Froquet R, Marchetti A, Bergeret E *et al.* Specific host genes required for the killing of Klebsiella bacteria by phagocytes. *Cell Microbiol* 2006; **8**: 139–148.

111 Le Coadic M, Froquet R, Lima WC, Dias M, Marchetti A, Cosson P. Phg1/TM9 proteins control intracellular killing of bacteria by determining cellular levels of the Kil1 sulfotransferase in Dictyostelium. *PLoS One* 2013; **8**: e53259.

112 Cosson P, Lima WC. Intracellular killing of bacteria: is Dictyostelium a model macrophage or an alien? *Cell Microbiol* 2014; **16**: 816–823.

113 Peleg-Grossman S, Volpin H, Levine A. Root hair curling and Rhizobium infection in Medicago truncatula are mediated by phosphatidylinositide-regulated endocytosis and reactive oxygen species. *J Exp Bot* 2007; **58**: 1637–1649.

114 Maruyama S, Kim E. A modern descendant of early green algal phagotrophs. *Curr Biol* 2013; **23**: 1081–1084.

115 Yoon HS, Muller KM, Sheath RG, Ott FD, Bhattacharya D. Defining the major lineages of red algae (Rhodophyta)1. *J Phycol* 2006; **42**: 482–492.

116 Yubuki N, Nakayama T, Inouye I. A UNIQUE LIFE CYCLE AND PERENNATION IN A COLORLESS CHRYSOPHYTE SPUMELLA SP.(1). *J Phycol* 2008; **44**: 164–172.

117 Sockett RE, Lambert C. Bdellovibrio as therapeutic agents: a predatory renaissance? *Nat Rev Microbiol* 2004; **2**: 669–675.

118 Qureshi MA, Ali RA. Spirulina platensis exposure enhances macrophage phagocytic function in cats. *Immunopharmacol Immunotoxicol* 1996; **18**: 457–463.

119 Panaro MA, Acquafredda A, Lisi S, Lofrumento DD, Mitolo V, Sisto M *et al.* Nitric oxide production by macrophages of dogs vaccinated with killed Leishmania infantum promastigotes. *Comp Immunol Microbiol Infect Dis* 2001; **24**: 187–195.

120 Stelzer G, Rosen N, Plaschkes I, Zimmerman S, Twik M, Fishilevich S *et al.* The GeneCards Suite: From Gene Data Mining to Disease Genome Sequence Analyses. *Curr Protoc Bioinformatics* 2016; **54**: 1.30.1-1.30.33.

121 Sarode SC, Sarode GS, Chuodhari S, Patil S. Non-cannibalistic tumor cells of oral squamous cell carcinoma can express phagocytic markers. Journal of Oral Pathology & Medicine. 2017; **46**: 327–331.

122 Schenker H, Büttner-Herold M, Fietkau R, Distel LV. Cell-in-cell structures are more potent predictors of outcome than senescence or apoptosis in head and neck squamous cell carcinomas. *Radiat Oncol* 2017; **12**: 21.

123 Fais S, Overholtzer M. Cell-in-cell phenomena in cancer. *Nat Rev Cancer* 2018; **18**: 758–766.

124 Erdman LK, Cosio G, Helmers AJ, Gowda DC, Grinstein S, Kain KC. CD36 and TLR interactions in inflammation and phagocytosis: implications for malaria. *J Immunol* 2009; **183**: 6452–6459.

125 Jain M. An overview on “cellular cannibalism” with special reference to oral squamous cell carcinoma. *Exp Oncol* 2015; **37**: 242–245.

126 Sun Q, Luo T, Ren Y, Florey O, Cibas ES, Hodgson L. Abstract SY29-03: Competition between tumor cells by entosis. *Cancer Res* 2014.https://aacrjournals.org/cancerres/article/74/19_Supplement/SY29-03/598693.

127 Purvanov V, Holst M, Khan J, Baarlink C, Grosse R. G-protein-coupled receptor signaling and polarized actin dynamics drive cell-in-cell invasion. *Elife* 2014; **3**. doi:10.7554/eLife.02786.

128 Hofmann A, Putz F, Büttner-Herold M, Hecht M, Fietkau R, Distel LV. Increase in non-professional phagocytosis during the progression of cell cycle. *PLoS One* 2021; **16**: e0246402.

129 Sun Q, Cibas ES, Huang H, Hodgson L, Overholtzer M. Induction of entosis by epithelial cadherin expression. *Cell Res* 2014; **24**: 1288–1298.

130 Paolillo R, Spinello I, Quaranta MT, Pasquini L, Pelosi E, Lo Coco F *et al.* Human TM9SF4 Is a New Gene Down-Regulated by Hypoxia and Involved in Cell Adhesion of Leukemic Cells. *PLoS One* 2015; **10**: e0126968.

131 Westphal A, Cheng W, Yu J, Grassl G, Krautkrämer M, Holst O *et al.* Lysosomal trafficking regulator Lyst links membrane trafficking to toll-like receptor–mediated inflammatory responses. *J Exp Med* 2017; **214**: 227–244.

132 Schutt B. *Cannibalism: A Perfectly Natural History*. Algonquin Books, 2018.

133 Vilarinho EC, Fernandes OA, Hunt TE, Caixeta DF. Movement of" Spodoptera frugiperda" adults (Lepidoptera: Noctuidae) in Maize in Brazil. *Fla Entomol* 2011.https://www.jstor.org/stable/41336431.

134 Yang Y-Q, Li J-C. Progress of research in cell-in-cell phenomena. *Anat Rec*  2012; **295**: 372–377.

135 Alfarouk KO, Muddathir AK, Shayoub MEA. Tumor acidity as evolutionary spite. *Cancers*  2011; **3**: 408–414.

136 Atkins EL, Banker R, Butler CG, Cale GH, Cale GH. The hive and the honey bee. *Dadant and Sons, Hamilton* 1975.

137 Abdu Y, Maniscalco C, Heddleston JM, Chew T-L, Nance J. Developmentally programmed germ cell remodelling by endodermal cell cannibalism. *Nat Cell Biol* 2016; **18**: 1302–1310.

138 Schafer DP, Lehrman EK, Kautzman AG, Koyama R, Mardinly AR, Yamasaki R *et al.* Microglia sculpt postnatal neural circuits in an activity and complement-dependent manner. *Neuron* 2012; **74**: 691–705.

139 Via S. Cannibalism facilitates the use of a novel environment in the flour beetle, Tribolium castaneum. *Heredity*  1999; **82 ( Pt 3)**: 267–275.

140 Gast CE, Silk AD, Zarour L, Riegler L, Burkhart JG, Gustafson KT *et al.* Cell fusion potentiates tumor heterogeneity and reveals circulating hybrid cells that correlate with stage and survival. *Sci Adv* 2018; **4**: eaat7828.

141 Krajcovic M, Overholtzer M. Mechanisms of ploidy increase in human cancers: a new role for cell cannibalism. *Cancer Res* 2012; **72**: 1596–1601.

142 Sarode SC, Sarode GS. Neutrophil-tumor cell cannibalism in oral squamous cell carcinoma. *J Oral Pathol Med* 2014; **43**: 454–458.

143 Sarode SC, Sarode GS. Cellular cannibalism in central and peripheral giant cell granuloma of the oral cavity can predict biological behavior of the lesion. *J Oral Pathol Med* 2014; **43**: 459–463.

144 Powell AE, Anderson EC, Davies PS, Silk AD, Pelz C, Impey S *et al.* Fusion between Intestinal epithelial cells and macrophages in a cancer context results in nuclear reprogramming. *Cancer Res* 2011; **71**: 1497–1505.

145 Lindström A, Midtbö K, Arnesson L-G, Garvin S, Shabo I. Fusion between M2-macrophages and cancer cells results in a subpopulation of radioresistant cells with enhanced DNA-repair capacity. *Oncotarget* 2017; **8**: 51370–51386.

146 Vogel D, Dussutour A. Direct transfer of learned behaviour via cell fusion in non-neural organisms. *Proc Biol Sci* 2016; **283**. doi:10.1098/rspb.2016.2382.

147 Xia P, Wang S, Guo Z, Yao X. Emperipolesis, entosis and beyond: dance with fate. *Cell Res* 2008; **18**: 705–707.

148 Radosević K, van Leeuwen AM, Segers-Nolten IM, Figdor CG, de Grooth BG, Greve J. Occurrence and a possible mechanism of penetration of natural killer cells into K562 target cells during the cytotoxic interaction. *Cytometry* 1995; **20**: 273–280.

149 Gupta N, Jadhav K, Shah V. Emperipolesis, entosis and cell cannibalism: Demystifying the cloud. *J Oral Maxillofac Pathol* 2017; **21**: 92–98.

150 Vilaca A. Relations between Funerary Cannibalism and Warfare Cannibalism: The Question of Predation. *Ethnos* 2000; **65**: 83–106.

151 Borensztejn K, Tyrna P, Gaweł AM, Dziuba I, Wojcik C, Bialy LP *et al.* Classification of Cell-in-Cell Structures: Different Phenomena with Similar Appearance. *Cells* 2021; **10**. doi:10.3390/cells10102569.

152 Garcı́a-Olmo D, Garcı́a-Olmo DC, Ontañón J, Martinez E. Horizontal transfer of DNA and the “genometastasis hypothesis.” *Blood* 2000; **95**: 724–725.

153 Bergsmedh A, Szeles A, Spetz A-L, Holmgren L. Loss of the p21Cip1/Waf1 cyclin kinase inhibitor results in propagation of horizontally transferred DNA. *Cancer Res* 2002; **62**: 575–579.

154 Bergsmedh A, Ehnfors J, Kawane K, Motoyama N, Nagata S, Holmgren L. DNase II and the Chk2 DNA damage pathway form a genetic barrier blocking replication of horizontally transferred DNA. *Mol Cancer Res* 2006; **4**: 187–195.

155 Ni C, Chen Y, Zeng M, Pei R, Du Y, Tang L *et al.* In-cell infection: a novel pathway for Epstein-Barr virus infection mediated by cell-in-cell structures. *Cell Res* 2015; **25**: 785–800.
