# Supplementary material for: Cell-in-cell phenomena across the tree of life
Source: Sci Rep. 2024 Mar 29;14:7535. doi: 10.1038/s41598-024-57528-7 (PMC10980697; doi:10.1038/s41598-024-57528-7)
Supplement: Supplementary file 3 — Supplementary Figure 1. [file 41598_2024_57528_MOESM3_ESM.docx]

**Supplementary Figure. PRISMA flow chart leading to the selection of 115 papers included in this review.**

**Identification of studies via databases**

Records identified from:

- Arizona State University library search engine (n = 352)
- Citation searches (n ~ 156)

Records removed before screening:

- Non-English records (n = 3)
- Reviews (n = 102)
- Newspaper articles (n = 4)
- Book chapters (n = 0)
- Conference proceedings (n = 0)
- Theses (n = 62)

Identification

Records for initial assessment

(n = 337)

Records excluded by reading only their title

(n = 0):

- Irrelevant titles (n = 0)

Screening

Records excluded (n = 222):

- Irrelevant topic (n = 38)
- Directed us to other possibly relevant articles (n = 97)
- No information on cell-in-cell phenomena in specific taxa/species (n = 41)
- Unknown whether the engulfed/host cell dies or both remain alive (n = 45)
- Removed Provora as the taxon was not found in timetree.org (n = 1)

Records for assessment of abstract or full text

(n = 337)

Studies included in review

(n = 115)

Included
